# Applications of Raman spectroscopy in the post-mortem interval estimation: a systematic literature review

**DOI:** 10.1007/s00414-026-03738-9

**Published:** 2026-03-02

**Authors:** Sara Sablone, Andrea Nicola Cardinale, Antonio Di Lorenzo, Giuseppe Strisciullo, Kanar Alkass, Henrik Druid, Valentina Mussi

**Affiliations:** 1 Section of Legal Medicine, Interdisciplinary Department of Medicine, University of Bari, Bari Policlinico Hospital, Bari, Italy; 2 Interdisciplinary Department of Medicine, University of Bari, Bari Policlinico Hospital, Bari, Italy; 3 Section of Legal Medicine, Forensic Toxicology Laboratory, Interdisciplinary Department of Medicine, University of Bari, Bari Policlinico Hospital, Bari, Italy; 4 Department of Oncology-Pathology, Karolinska Institutet, Stockholm, Sweden; 5 IMM CNR, Institute of Microelectronics and Microsystems, National Research Council, Rome, Italy

**Keywords:** Raman spectroscopy, Post-mortem interval, Burial time, Forensic pathology, Human skeletal remains, Forensic anthropology.

## Abstract

Post-mortem interval (PMI) estimation remains a challenge for forensic pathologists, thus representing a hot topic in the research of forensic sciences. Over recent years, new methods have been proposed based on the analysis of various human biological matrices and changes in their post-mortem biochemical markers (BPMs) concentrations. Raman spectroscopy shows great promise in PMI assessment thanks to its ability to analyze complex organic materials and provide a unique fingerprint of substances. Following PRISMA guidelines, this systematic review addresses the PICO question: “In human cadavers/remains, what is the role of Raman spectroscopy, on what biological matrices, and with what analytical techniques is it currently applied for post-mortem interval estimation?“. The selected five papers reviewed point out that the applications of Raman spectroscopy in PMI estimation are now widespread in forensic anthropology, moving from the study of evolving chemical alterations of human skeletal remains over time to exploring their correlation with burial duration or PMI. The most frequently evaluated parameters were phosphate (an inorganic component of the bone/tooth matrix) and the progressive decrease in the organic bands’ peak intensity over PMI, with principal component analysis (PCA) being the preferred multivariate statistical analysis to improve the interpretation of Raman spectral data. Although Raman spectroscopy has many analytical and practical advantages, being non-destructive, rapid, and available as an hand-held device, its implementation in forensic routine casework remains limited and can only be expected to be useful in cases with longer PMI.

## Introduction

The evaluation of the time elapsed since death, defined as post-mortem interval (PMI), represents one of the main challenges for the medico-legal examiners due to its significant implications in criminal and civil contexts [1–6]. Classical methods for PMI estimation rely on body cooling using Henssge´s nomogram [7] (which considers rectal and ambient temperature and body weight), mechanical and electromuscular reactivity, and simple changes in livores and lividity [4]. However, these methods are limited to the early post-mortem phase, usually within the first 24 h, after which rectal temperature equals the environmental temperature. Among alternative approaches, forensic entomology - despite its well-known limitations - represents a method that proved to satisfy Daubert’s criteria for admissibility in court as scientific evidence (estability/falsifiability; peer review and publication; known error rate and standards/controls; existence of standards; general acceptance) [8]. Due to the need for more precise PMI estimation over 24 h, existing methodologies have been further developed, including studies on skeletal muscle and pupil excitability, and new methods have been proposed, such as thanatochemistry and metabolomics [9–12].

In particular, thanatochemistry investigates time-dependent variations of single metabolites in a biological matrix, revealing potential reproducible biochemical patterns, especially for hypoxanthine (Hx) and potassium (K⁺) in vitreous humor [13, 14]. This approach demonstrated reliable PMI prediction up to 100 h with regression models with acceptable standard deviations [15, 16]. In addition, in an effort to improve estimation accuracy, recent studies have applied AI and machine learning tools (additive models and Support Vector Machines) to thanatochemical and metabolomic datasets [17, 18].

In recent years, spectroscopic techniques such as Nuclear Magnetic Resonance (NMR), Fourier Transform Infrared (FTIR), and Raman spectroscopy have been introduced as innovative tools for PMI estimation [19]. These methods reveal structural and chemical modifications in hard and soft tissues reflecting biochemical changes after death.

Among these, Raman spectroscopy is a non-invasive analytical technique that provides information about molecular vibrations and consequently the structure of analyzed samples [20].

From a physical point of view, when a monochromatic light impinges on a sample, some may be absorbed while much passes unchanged through it, depending on the light wavelength and the nature of the sample. A small fraction (~ 0.1%) is elastically scattered as light showing the same frequency as the incident light (Rayleigh scattering). An even smaller fraction of the incident light (~ 1 photon in 10^6^ or 10^7^) is scattered inelastically. One such inelastic diffusion mechanism is Raman scattering, where the change in photon energy is associated with the excitation of specific molecular vibrations, which can be considered a fingerprint uniquely identifying the substance [20].

Raman spectroscopy offers several advantages as a complementary method for forensic evidence analysis [21]: (i) it is a highly selective determination technique that acts as a molecular fingerprint containing unique, reproducible, detailed features; (ii) it can analyze any optically accessible sample (biological, organic, or inorganic; in solid, liquid, or gas phase); (iii) the sample does not require pre-scan preparations; (iv) the scan is non-invasive and non-destructive; (v) it has completed its transformation into a fully portable technique for on-field analysis.

Moreover, thanks to the large number of peer-reviewed publications, the ability to assess error rates, its wide acceptance within the scientific community, and the existence of established standard procedures, Raman spectroscopy satisfies Daubert standards and has been recognized as admissible scientific evidence in the U.S. courts [8, 22].

Raman spectroscopy has been applied in forensic contexts for the identification of biofluids, explosives, gunshot residues, and drugs [23–26]. Specifically, unique spectroscopic signatures of biofluids detected by Raman spectroscopy allowed to identify blood, saliva, sweat, semen, and vaginal fluids, but also to determine sex, age, and race of a suspect and the bloodstain age [27–31].

Although it is not a new technique and its use is now widespread in forensic sciences, Raman spectroscopy applications are still little known among forensic pathologists and not commonly used for investigations of their specific relevance.

To the best of our knowledge, no recent literature reviews have specifically addressed the role of Raman spectroscopy in PMI estimation.

Thus, following the PRISMA guidelines, we conducted a comprehensive and critical review of the relevant scientific literature to establish the state-of-art and inform the ongoing laboratory research about the possible Raman applications for the PMI assessment, with a focus on methodological strengths, limitations, and forensic applicability of this technique.

## Materials and methods

This review followed the PRISMA (Preferred Reporting Items for Systematic Reviews and Meta-Analyses) protocol [32]. The articles were selected with no time limits from PubMed, Scopus, Web of Science, and Cochrane Library databases and by the query: (“Raman” or “Raman spectroscopy”) AND (“postmortem interval” or “post-mortem interval” or “PMI” or “time of death” or “time since death” or “time from death” or “time after death”). This query was focused on the following PICO (Population, Intervention, Comparison and Outcome) question [33]: “In human cadavers/remains, what is the role of Raman spectroscopy, on what biological matrices, and with what analytical techniques is it currently applied for the post-mortem interval estimation?”. To address this question, articles were excluded if they: (i) were not published in English; (ii) had non-eligible formats (such as reviews not including research studies, case reports, or conference proceedings); (iii) were not of forensic interest; (iv) did not focus on Raman spectroscopy and PMI (or exclusively focused on burial duration, without correlation with PMI). Potentially relevant studies that did not emerge in the main search were also identified from the references of other articles (mainly review articles). Articles were selected according to a standardized procedure, by screening the title, the abstract, and, finally, the full text. Eligibility was assessed independently by all authors, and any disagreements were resolved by consensus. Data were extracted from each study and organized into a table, pointing out detailed information about types of study, types and size of biological matrices, environmental exposure conditions of biological matrices, pre-analytical and analytical processes, detection methods, statistical analysis (if available), and the main results.

## Results

### Data extraction

**Fig. 1:**
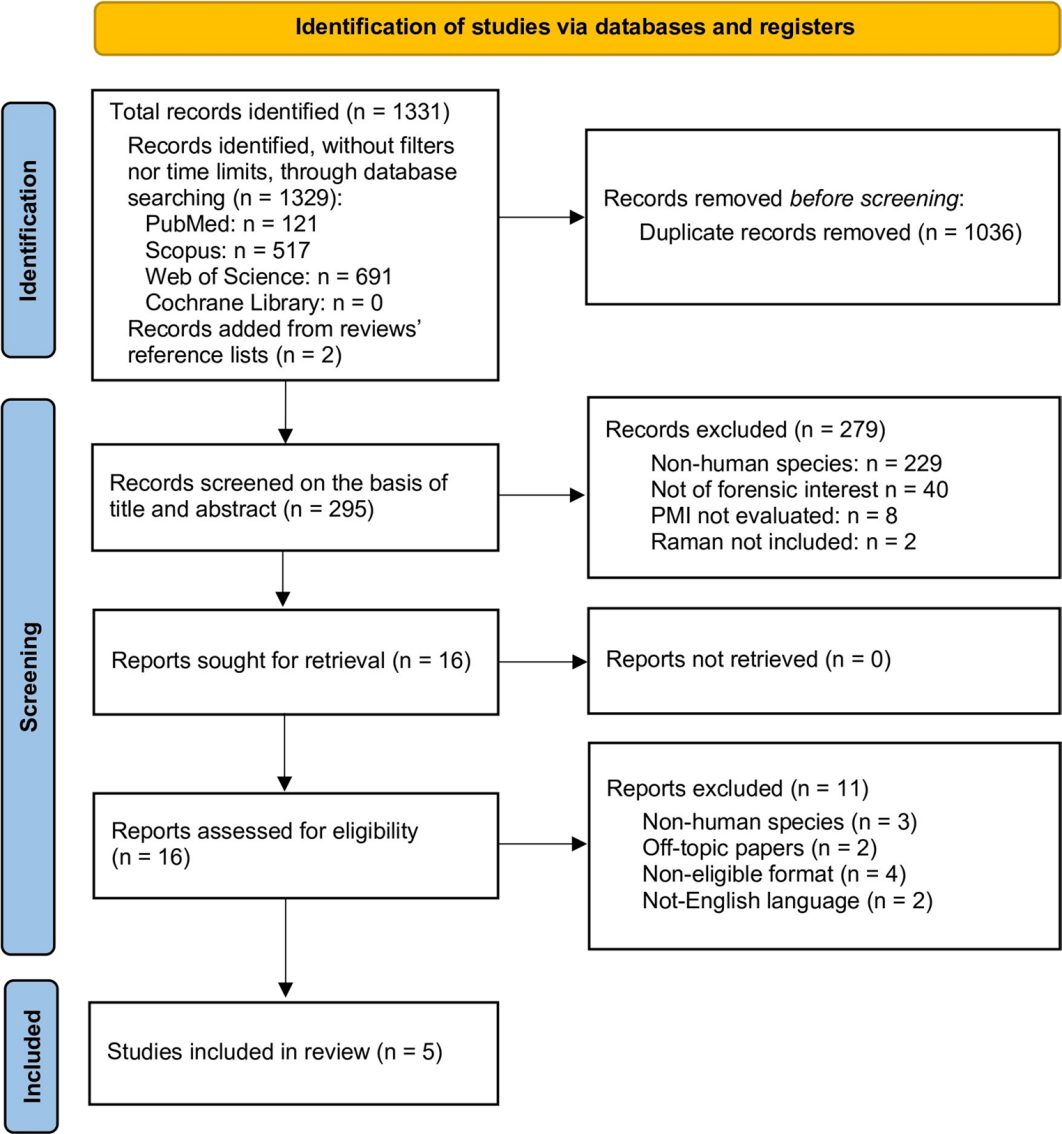
PRISMA Flow Chart

A total of 1331 articles were retrieved from the database search and by consulting their References’ sections. After duplicate removal, 295 remaining articles underwent title and abstract screening, leading to the exclusion of an additional 279 entries. The remaining 16 papers were retrieved for full-text review. Out of 16 articles, 5 were included in the review. The remaining eleven articles were excluded due to non-human species reference (3 articles), off-topic evaluation (2 articles), inadequate article type (4 articles), English language not available (2 articles). The article selection process is outlined in Fig. 1.

Table 1 provides the details of the 5 selected articles. They are all original research articles, performed in Italy, Austria, USA, Spain, and France, respectively.

**Table 1:** Information included in the papers selected for review

Authors and Year of publication	Title	Type of article	Place the study was performed
Bertoluzza A et al. 1997 [34]	Preliminary Results in Dating Human Skeletal Remains by Raman Spectroscopy	Research Article	Italy
Woess C et al. 2017 [35]	Assessing various Infrared (IR) microscopic imaging techniques for post-mortem interval evaluation of human skeletal remains	Research Article	Austria
Baide A et al. 2020 [36]	Non-invasive post-mortem interval diagnostic using a hand-held Raman spectrometer	Research Article	United States of America (Texas)
Ortiz-Herrero L et al. 2021 [37]	Estimation of the post-mortem interval of human skeletal remains using Raman spectroscopy and chemometrics	Research article	Spain
Falgayrac G et al. 2022 [38]	Critical aspects of Raman spectroscopy as a tool for postmortem interval estimation	Research Article	France

### Biological matrices and donor corpses’ characteristics

The selected studies examined different biological matrices’ types and sizes (Table 2). Bertoluzza et al. [34] and Baide et al. [36] studied dental remains by examining enamel, with Bertoluzza [34] also considering dentine. In contrast, the other studies [35, 37, 38] focused on bone samples, with Woess [35] specifically analyzing femurs and Falgayrac [38] examining ribs.

Different sample sizes were observed in the selected studies. Notably, Ortiz-Herrero et al. [37] analyzed more than 12 samples, whereas Woess et al. [35] and Falgayrac et al. [38] reported sample sizes between 10 and 12. The studies by Bertoluzza et al. [34] and Woess et al. [35] included only six samples, with Bertoluzza et al. [34] and Falgayrac et al. [38] being the only authors to apply exclusion criteria for the samples examined (excluding teeth affected by caries and tartar (dental calculus) and selecting only fresh bones without known bone pathologies, respectively).

The age of donor corpses was reported in two studies [36, 38], revealing that the latter includes significantly older deceased subjects. Furthermore, the studies by Baide et al. [36] and Ortiz-Herrero [37] are the only ones to distinguish between sexes, showing a general predominance of male donor corpses. Despite the small sample size, the study by Falgayrac et al. [38] was the only one to report detailed biological and demographic information, including diseases, cause of death, and ethnicity, thereby emphasizing inter-individual variability.

**Table 2:** Information about biological matrices and donor corpses’ characteristics

Biological matrices	Sample size and donor corpse personal characteristics (if available)	Exclusion criteria in sampling selection	Declared limitations
Teeth (molars and canine) dentine and enamel [34]	6 samples: - 5 ancient interred molar teeth; - 1 modern deciduous canine of an infant.	Samples affected by caries or tartar	N.A.
Bones (upper and mid third of the femur) [35]	6 samples: - 2 archeological remains from medieval times; - 4 forensic bone samples.	N.A.	N.A.
Teeth (incisors) enamel [36]	10 samples from 8 non-buried human cadavers: - 6 males and 2 females; - mean age: 64 years.	N.A.	Following interfering factors are known: - temperature: initial season of placement differs between subjects; - insect activity: prevention attempt by using metal cafes wrapped in chicken wire placed on the top of the human cadaver; - humidity; - embalming.
Bones (left tibia diaphyses) [37]	53 samples: - from niches of a burial site in Granada: 50% males and 50% females; - from niches of a burial site in Córdoba: 60% males and 40% females.	N.A.	N.A.
Bones (R1 and R4) [38]	12 (interred) samples from 6 subjects: - Age: between 72 and 92 (mean: 83.5 (+/- 7,1); - Diseases/conditions: a. none (n: 2/6); b. coxarthrosis, knee surgery, osteonecrosis of femoral head (n: 1/6); c. heart prosthetic stent, vascular issues (n: 1/6), Alzheimer’s disease (n: 1/6); d. Hypertension (n: 1/6); e. AML, Alcoholism, smoking (n: 1/6); - Cause of death: hearth attack (n: 6/6); - Race: Caucasian (6/6).	Presence of known bone pathologies. Fixation by any protocol undergone by bones.	Limited number of bone donors (only 6) due to limited accessibility to human specimens. Samples deriving only from old subjects.

*N.A.* Not Applicable, *R1-R4 *Ribs 1–4, *AML *Acute Myeloid Leukemia

### Environmental exposure conditions of biological matrices and PMI/burial time assessed

As pointed out in Table 3, four papers specified the environmental conditions to which the studied biological matrices were exposed before analysis [35–38]. Namely, they specified the ambient temperature at which samples were stored. Furthermore, Baide et al. [36] and Ortiz-Herrero et al. [37] reported annual precipitation, while only Falgayrac et al. [38] explicitly stated humidity levels. Despite its potential influence on spectral variation over PMI, soil characteristics were specified only by Ortiz-Herrero et al. [37] and Falgayrac et al. [38]. Interestingly, Ortiz-Herrero et al. [37] were the only researchers to perform their study in two different geographical regions with distinct environmental conditions.

The burial time/PMI assessed is specified in all studies. A wide variation in the timespan analyzed was evident, ranging from a few days [34–36] or months [38] to tens [37] or thousands of years [34, 35]. Specifically, Bertoluzza et al. [34] and Falgayrac et al. [38] focus on burial time, with Falgayrac [38] providing a precise estimation of this parameter due to the study design, which allows for a controlled observation of the actual burial time. In contrast, Woess et al., Ortiz-Herrero et al., and Baide et al. [35–37] refer to the same parameter as PMI, which is a more general term used to describe the time since death. In particular, in the studies reviewed all authors report the PMI or burial time for each sample, except for Baide et al. [36], who only specify the time span analyzed.

Interestingly, Woess’s study [35] is the only one to express findings in terms of Accumulated Degree Days (ADD), stating that a PMI of 27 to 266 days corresponds to ADD values ranging from 421 to 4,993. This approach highlights the influence of environmental factors, particularly temperature, on the decomposition process. In fact, ADD is a metric that quantifies the effect of temperature over time on biological processes like decomposition. It is a metric used to estimate the decomposition rate of a body by considering both temperature and time, operating on the principle that higher temperatures accelerate decomposition, while lower temperatures slow it down. ADD is calculated by summing the differences between the daily average temperatures and a base temperature threshold, below which decomposition is negligible, adopting the formula $\:ADD={\sum\:}_{i=1}^{n}\left(Ti-Tbase\right)$, where $\:Ti$ is the daily average temperature (°C) and $\:Tbase\:$is the base temperature (°C), often set between 0 °C and 10 °C [39]. 

**Table 3:** Information about environmental exposure conditions of biological matrices and PMI/burial time assessed

Temperature (environmental conditions)	Annual precipitations	Soil characteristics	PMI/burial time analyzed*
N.A.[34]	N.A.	N.A.	*Age of burial* *dated by*: ^◆^*radiometric (*^*14*^*C) or °archeological methods*: 0 years*°* 150 years^◆^ 650 years*°* 1700–1800 years*°* 2500–2600 years*°* 5800–6300 years^◆^
Room temperature [35]	N.A.	N.A.	*PMI* 1 day*°* (forensic) 3 years*°* (forensic) 25 years*°* (forensic) 85 years*°* (forensic) 650–870 years^◆^ (archaeologic) 1030–1260 years^◆^ (archaeologic) *°missing persons or* ^◆^*archeological remains dated by radiocarbon methods*.
Mean temperature: 19,4 °C. [36]	857 mm	N.A.	*PMI* 27 days – 266 days, obtained by periodically re-analyzing the samples over the time through the spectrometer. The study does not provide the specific PMI of every sample. The observed ADD values ranged from 421 to 4993.
Mean temperatures: 17,8 °C (Córdoba) and 15,5 °C (Granada). [37]	612 mm (Córdoba) and 450 mm (Granada).	Human remains had no contact with the soil. Instead, they were buried in niches, and therefore were less prone to fungal and bacterial attacks.	*PMI (years)* 15, 15, 17, 18, 19, 20, 20, 21, 22, 22, 24, 25, 25, 26, 27, 28, 29, 29, 30, 31, 31, 31, 33, 33, 33, 34, 35, 36, 37, 38, 39, 41, 42, 43, 44, 44, 44, 45, 46, 48, 48, 49, 50, 51, 51, 51, 53, 53, 57, 58, 65, 87.
Temperature range: - Mean: 10,5 ± 4,7 - Max: 14,4 ± 5,4 - Min: 7,0 ± 4,2 [38]	Humidity: 79,5% ± 6,8%	The environment was under cover. Soil type: clay. Soil pH: 6,8.	*Burial time* 1 month, 2 months, 3 months, 4 months, 5 months, 6 months, 7 months, 8 months, 9 months, 10 months, 11 months, 12 months. The samples were harvested on the day of death. Then, the procedure of sampling and collection was applied each month for 12 months.

*N.A.* Not Applicable, *PMI *Post-Mortem Interval, *ADD *Accumulated Degree Days

* In the selected studies, “burial time” and “PMI” are interchangeable when the latter has such high values that any difference between them is infinitesimal

### Pre-analytical sample treatment and Raman spectra acquisition

Two studies [37, 38] applied a preliminary sample treatment before Raman spectroscopy acquisition (Table 4). In the first case, samples were bathed in sodium hypochlorite and scrubbed to remove fungal and bacterial contamination. Moreover, photobleaching – i.e., the irreversible destruction of a luminous molecule after absorbing too much light energy – was also employed to minimize the fluorescent background and improve the accuracy of spectra acquisition. In the second case, the ribs were cleaned of the surrounding flesh and dissected into fragments for Raman spectroscopy analysis. After the first round of Raman recording (T0), ribs were replaced in the burial niches and the measurement procedure was repeated every month for 12 months.

The resolution of Raman acquisition was specified in three studies [34, 35, 37], ranging from 6 cm⁻¹ [35] to 3.24 cm⁻¹ [37]. While the acquisition time was explicitly stated only by Falgayrac et al. [38], the spectral range analyzed was specified only by Woess et al. [35] and Ortiz-Herrero et al. [37], with the latter exploring a wider spectral range.

**Table 4:** Information about detection methods, pre-analytical sample treatment, and Raman spectra acquisition

Detection methods	Pre-analytical phase	Spectral range and resolution
FT-Raman (Bruker IFS 66 Instrument with FRA 106 Raman attachment). 4 different positions of spectra collection. [34]	N.A.	500 scans at spectral resolution of 4 cm^− 1^
Raman imaging (WITec ALPHA300R microscope). For excitation in near-infrared: Toptica XTRA laser (wavelenght of 785 nm; power of 15 mW). Parameters to reduce measurement times for a single scan. Raman imaging (Chemi maps and MIAs) and PCA analyses were performed. [35]	N.A.	Spectral range analyzed: 0 cm^− 1^ to 1776 cm^− 1^. Spectral resolution: 6 cm^− 1^.
Raman spectra collection with a hand-held portable Agilent Resolve spectrometer, with the following parameters: acquisition time of 1 s; power of 495 mW. Import of spectra into MATLAB for ANOVA (Analysis of variance). [36]	N.A.	N.A.
Use of a NRS-5100 Dispersive Raman Spectrometer with a confocal microscope (x100) and a 785 nm excitation wavelength laser (power of 53,1 mW). [37]	Water bath with sodium hypochlorite and brushing were performed to eliminate fungal and bacterial contamination. Brushed bones were stored inside wooden boxes at a constant temperature (20–25 °C) for conservation. Prior to spectra acquisition, photobleaching was performed for 3 min to reduce bone fluorescence and minimize background interference.	Scan range: 3200–200 cm^− 1^. Spectral resolution: 3,24 cm^− 1^.
Raman spectra acquired through LabRAM HR800 Raman microspectrometer with DuoScan technology and a 785 nm laser diode. For each ribs, 4 spectra were acquired. [38]	Flesh around ribs was removed. Bone section for Raman spectroscopy was performed. Covering with neutral wax of the rib holes resulted from bone transversal cuts.After first Raman registration performed at death (T_0_), each pair of ribs was put back on plastic trays filled with clay soil typical of northern France (the ribs were covered by approximately 1 cm of soil) and placed outside and under cover to protect them from scavengers and rain. The measurement procedure was repeated each month for 12 months.	Acquisition time: 30 s.

*N.A.* Not Applicable, *MIA *Multivariate Imaging Analysis, *FT-Raman Spectroscopy *Fourier Transform Raman Spectroscopy, *PCA *Principal Component Analysis, *ANOVA *Analysis of Variance

### Raman spectroscopy bands and peaks analyzed

Table 5 summarizes the most informative bands and peaks analyzed in the selected articles for burial time/PMI estimation.

#### Phosphate bands

The band attributed to the A₁ symmetric stretching mode of phosphate (ν₁ PO₄³⁻), located around 957–960 cm⁻¹, was the most frequently analyzed, appearing in the studies conducted by Bertoluzza et al. [34], Baide et al. [36], Ortiz-Herrero et al. [37], and Falgayrac et al. [38]. Other significant peaks were identified at 427–433 cm^− 1^ and 577–590 cm^− 1^ [36, 38], and were attributed to different vibrational configurations.

Other phosphate-related bands received less attention. Notably, the study by Ortiz-Herrero et al. [37] identified slightly wider intervals for this mineral component. Specifically, the asymmetric bending mode ν₄ (PO₄³⁻), with a band around 600 cm⁻¹, was considered by Baide et al. (609 cm⁻¹) [36], Falgayrac et al. (590 cm⁻¹) [38], and Woess et al. [35], with the latter not specifying the exact position in cm⁻¹. Additionally, Baide et al. [36] and Falgayrac et al. [38] examined the band around 430 cm⁻¹, corresponding to the symmetric bending mode ν₂ PO₄³⁻. Baide et al. [36] also reported an additional phosphate band at 587 cm⁻¹, close to the asymmetric bending mode T₂ (ν₄ (PO₄³⁻)).

#### Carbonate bands

All included studies consider carbonate-related peaks, yet with considerable heterogeneity in their attributions. The only consistently reported carbonate peak was found around 1070 cm⁻¹, attributed to the symmetric stretching mode ν₁ CO₃²⁻ and observed by Baide et al. [36] and Falgayrac et al. [38]. In contrast, Bertoluzza et al. [34] analyzed the ν₂ CH₂ peak at 2941 cm⁻¹, while Woess et al. [35] examine the B-type carbonate band at 756 cm⁻¹, corresponding to the out-of-plane bending mode ν₂. Even in this case, Ortiz-Herrero et al. [37] identified slightly wider intervals for this mineral component and reported a carbonate peak at 1979 cm⁻¹. Interestingly, Baide et al. [36] identified two carbonate bands: in addition to the previously mentioned 1070 cm⁻¹ (ν₁ CO₃²⁻), a second peak at 1402 cm⁻¹ was assigned to the asymmetric stretching mode ν₃ CO₃²⁻. The analysis of both the ν₁ symmetric stretching vibration band and the ν₃ asymmetric stretching band provides insights into bone diagenesis. Indeed, it reveals shifts in the carbonate bands’ position, intensity, or relative ratio, which aresensitive to changes in carbonate incorporation within the bone mineral component.

#### Collagen amide bands

As far as collagen is concerned, the protein’s large and complex molecular structure accounts for a wide range of bands. Collagen-proline and hydroxyproline were shown to scatter light at 851 cm^− 1^, 917 cm^− 1^, and within a narrow range between 873 and 880 cm^− 1^ [37]. A 1001–1003 cm^− 1^ scattering band was also attributed to phenylalanine [37].

Specifically, the amide bands of collagen were considered in three studies. The Amide I band (1600–1700 cm⁻¹), associated with C = O (carbonyl) stretching, was analyzed by Woess et al. [35] and Falgayrac et al. [38], with the latter specifically identifying a peak at 1670 cm⁻¹. The Amide III band, associated with vibrational contributions from C–N stretching and N–H bending, was reported by Baide et al. [36] (1243 cm⁻¹) and Falgayrac et al. [38] (1260 cm⁻¹). Notably, none of the reviewed studies investigated the Amide II band (1480–1575 cm⁻¹).

#### Other Collagen-Related bands

Ortiz-Herrero et al. [37] and Falgayrac et al. [38] addressed collagen bands but focused on different spectral features. Ortiz-Herrero et al. [37] examined bands at 1675 cm⁻¹ and 1645 cm⁻¹, while Falgayrac et al. [38] considered a band at 1450 cm⁻¹, attributed to collagen’s CH₂ groups. This band was near the “CH” band analyzed by Baide et al. (1454 cm⁻¹) [36], linked to CH-bending of methylene groups. In constrast, Bertoluzza et al. [34] examined the band attributed to symmetric stretching vibrations of methylene (v_s_ CH₂), observed approximately at 2941 cm⁻¹. Baide et al. [36] also investigated the NH group band, assigning it to a peak at 1402 cm⁻¹, whereas Woess et al. [35] examined an “α-helix” band located at 1272 cm⁻¹. Additional bands were described around 1295 cm^− 1^ for phospholipids [37].

#### Indices based on peak ratios

Several studies [34, 35, 37] developed specific indices based on peak ratios to examine how these ratios vary with the PMI. Bertoluzza et al. [34] and Woess et al. [35] primarily focused on ratios involving bands attributed to organic and inorganic substances. These ratios showed either an increasing or decreasing trend with the PMI, in line with the behavior of organic and inorganic components over time, resulting in either a positive or negative relationship depending on which component is in the numerator or denominator. In Bertoluzza’s research [34], the numerator featured the organic component, which refers to a peak at 2941 cm⁻¹ (I_2941_) in Raman spectra, while the denominator is the well-known 960 cm^− 1^ band (I_960_), corresponding to the inorganic v_1_ (PO_4_)^3−^ component, resulting in the I_2941_/I_960_ index, which showed to decrease with time. In contrast, Woess’s ratio [35], with the inorganic v_4_ (PO_4_)^3−^ band in the numerator and the Amide I band in the denominator, showed an increase with the PMI. Due to the absence of quantitative indicators (e.g., Mean Absolute Error, Root Mean Square Error, or R²), the PMI estimation accuracy of the abovementioned ratios cannot be compared.Unlike the previous approaches, Ortiz-Herrero et al. [37] introduced a variety of different indices and ratios. One of these – the so-called “mineral-to-matrix ratio” (1605 cm^− 1^/1720 cm^− 1^), compares organic and inorganic components, similarly to the aforementioned indices. Instead, others focused on entirely different parameters, such as the Carbonate-to-Phosphate ratio (1979 cm^− 1^/960 cm^− 1^), the collagen crosslinking ratio (1675 cm^− 1^/1645 cm^− 1^), and mineral crystallinity, which is calculated as the inverse of the v_1_ PO_4_^3−^ band width.

**Table 5:** Information about statistical analysis, peaks, and major findings

Statistical analysis	Peaks and parameters analyzed	Reviewed articles’ main findings
None [34]	Bands between 3000 and 1200 cm^− 1^ are mainly attributed to proteinaceous component of tooth (most intense peak: 2941 cm^− 1^ = v_s_CH_2_). Bands between 1200 and 200 cm^− 1^ are mainly attributed to inorganic component of teeth (most intense peak: 960 cm^− 1^ = v_1_(PO_4_)^3−^).	Progressive decrease of intensity of organic component of enamel with increasing burial time (PMI). The parameter to be considered is *I*_*2941*_*/I*_*960*_ (organic to inorganic component ratio) as a function to burial period. This ratio decreases with the progress of the burial time (mainly for the first years, less for longer periods).
Chemimaps/MIA and PCA analyses were performed on MIR, ATR and Raman imaging obtained spectra. [35]	*Raman imaging* - 756 cm^−1^ (B-type carbonate); - 957 cm^−1^ (HPO_4_); - 1272 cm^−1^ (protein α-elix). *Mineral content ratio* The parameter “v_4_(PO_4_)^3−^ peak/Amide I peak” has been calculated. *PCA analysis* 30 not-better-specified spectra were chosen.	*Raman imaging* Peak 756 cm^− 1^ (B-type carbonate) is not detectable after PMI of 3 years. Peak 957 cm^− 1^ (HPO_4_) shows only minimal differences over PMI. Peak 1272 cm^− 1^ (protein α-elix) is higher in forensic samples (i.e. samples with lower PMIs).*Mineral content ratio* v_4_(PO_4_)^3−^ peak/Amide I increased over PMI. During the age-related degradation, the mineral content ratio increases while the organic to mineral ratio decreases with time. *PCA analysis* Among the 30 spectra, the most descriptive information is included in the range 1700–300 cm^− 1^. When combined with PCA, the best segregation of forensic and archeological bone samples was obtained by Raman imaging (compared to ATR and IR reflection).
The AUC at the band 1402 cm^− 1^ at different PMI was analyzed through ANOVA to reveal P and F parameters (statistically significant difference for α = 0,05). Then, Tukey HSD test was applied to ANOVA-obtained data to compare different PMI groups. [36]	Following bands were analyzed in the spectrum: - 433 cm^−1^, 587 cm^−1^, 609 cm^−1^, 960 cm^−1^: PO_4_^3-^; - 1243 cm^−1^: amide III; - 1402 cm^−1^: NH; - 1454 cm^−1^: CH; - 1402 cm^−1^ and 1070 cm^−1^: CO_3_^2-^.	D22-43 showed weaker intensities; D64-84, D106-126 and D127-147 showed more intense bands. Amide III spectrum (1243 cm^− 1^) was more intense in D22-42 teeth, indicating a greater protein content on teeth surface. Only in D22-42 teeth following spectra were evident: 1110 cm^− 1^, 1180 cm^− 1^, 1353 cm^− 1^, 1513 cm^− 1^, 1532 cm^− 1^, and 1558 cm^− 1^. RS can identify accurately Stage I (early body decomposition, from D22 to D42), differentiating it from Stage II body decomposition stage (over D42) based on intensity of 1402 cm^− 1^ band (CO_3_^2−^). Similar spectra between D64-84 and older samples (D106-126 and D127-147) were observed but with an increase of bands at 1134 cm^− 1^ and 1180 cm^− 1^. D64-84 and D22-42 have singular vibrational fingerprints.
Data acquisition and analysis were performed by Spectra Manager II and WiRE 3 Renishaw. SIMCA 15.0.2 Umetrics was used for multivariate analysis. Through Hotelling’s T2 and distance-to-model plots of PCA, 6 out of 53 samples were excluded as outliers. The 47 remaining samples were subdivided in 33 samples (*training set*) and 14 samples (*test set*) by applying the Kennard-Stome-Algorithm. *Training set*: was used to elaborate a OPLSR model with 3LVs, obtaining RMSECV of 0,10 and R^2^ CV value of 0,89. *Test set*: application of the so-obtained OPLS model to the 14 remaining samples. Then, the so-obtained model with Latent Variables was assessed through following parameters: RMSECV, RMSEP, R^2^, and Q^2^. The *test set* highlighted an accuracy deviation of less than 30%. Accurate prediction of 71% of samples. The remaining 29% had a large deviation probably due to inter-individual variability affecting chemical components of bones. [37]	Carbonate-to-phosphate ratio (1979 cm^− 1^/960 cm^− 1^); mineral-to-matrix ratio (1605 cm^− 1^/1720 cm^− 1^), collagen crosslinking ratio (1675 cm^− 1^/1645 cm^− 1^) and mineral crystallinity (1/v_1_ PO_4_^3−^ band width) were calculated.	PMI greater than 15 years exhibited changes in v_2_(PO_4_^3−^) and v_4_(PO_4_^3−^) vibrational bands. PMI between 15 and 50 years showed changes in v_1_(PO_4_^3−^) bands. Modification in CH_2_, amide I and III bands were observed up to 87 years PMI. Raman combined with OPLS enables the visualization and interpretation of the modifications in spectra of human skeletons according to their PMI, offering the possibility of determining the PMI of human skeletal remains (≥ 15 years and ≤ 87 years) buried in similar conditions to those of the selected cemetery niches and in a geographic location with a Mediterranean climate.
ANOVA-simultaneous component analysis (ASCA) was used to evaluate the influence of factors on a determined set of recorded data. In particular, the ANOVA part of ASCA reveals factors with significant effect on data. The last step of the study was the SCA-part of ASCA, performed to detect the variability of data and identifying which Raman-band are more influenced by the factors X_A_ and X_B_. The factor X_A_ represents the source body effect, i.e., the variability due individual differences. Spectral variability influenced by X_A_ is observed in SC1 (phosphatic vibration v_1_PO_4_ at 960 cm^− 1^ and carbonate vibration v_1_CO_3_ at 1070 cm^− 1^) and SC2 (band of organic and mineral matrix, i.e., amide I at 1670 cm^− 1^ a, v_4_PO_4_ at 590 cm^− 1^ and v_2_PO_4_ at 430 cm^− 1^). The spectral variability influenced by the factor X_B_ (burial time) was also analyzed in SC1 and SC2. [38]	- 430 cm⁻¹ (phosphate v₂PO₄³⁻); - 590 cm⁻¹(phosphate v₄PO₄³⁻); - 960 cm⁻¹ (phosphate v₁PO₄³⁻); - 1070 cm⁻¹ (carbonate v₁CO₃²⁻); - 1260 cm⁻¹ (amide III); - 1450 cm⁻¹ (collagen’s CH_2_); - 1670 cm⁻¹ (amide I).	After 1000 permutations: - X_A_ (source body: intrinsic factor) explained 7,54% of variability (*p* < 0,001); - X_B_ (burial time: extrinsic factor) explained 15,66% of variability (*p* < 0,001); - exerted variance of 56,79% (*p* < 0,001). The main influence of X_A_ on SC1 was on intense band at 960 cm^− 1^ (phosphate band v_1_PO_4_) and a minor band at 1070 cm^− 1^ (carbon band). Influence of X_A_ on SC1 explains 72,26% of total variance, relating to mineral bands; influence of X_A_ on SC2 explains 16,83% of total variance relating to organic bands. Identified bands on SC2 include those related to organic and mineral matrix (amide I, 1670 cm^− 1^; CH_2_ collagen I, 1450 cm^− 1^; amide III, 1260 cm^− 1^; v_4_PO_4_. 590 cm^− 1^; v_2_PO_4_, 430 cm^− 1^). Influence of X_B_ on SC1 has a sinusoidal temporal trend, with strong contribution at 960 cm^− 1^ (carbonate band). The scores along SC2 show a general and irregular decrease over burial time, including positive scores between 0–5 months, negative scores between 5–12 months, with positive scores for mineral bands and negative for organic bands. Scores of SC2 represent 6,22% of total variance, while the decrease in mineral bands and increase in organic ones suggest a decrease in mineral/organic ratio. For the effects of X_B_ + X_AB_ (body/time interaction), both SC1 and SC2 had longitudinal scores plots, with the largest contribution showed by 960 cm^− 1^ and mineral and organic bands. The influence of X_A_ relates to inter-individual variability, but it is just half of the variability exerted by burial time.

*PMI* Post-Mortem Interval, *PCA *Principal Component Analysis, *ANOVA *Analysis Of Variance, *MIR *Mid Infrared Reflection, *D *day, *RS *Raman Spectroscopy, *OPLS *Orthogonal Projections to Latent Structures, *RMSE *Root Mean Square Error, *RMSECV *Root Mean Square Error of Cross-Validation, *RMSEP *Root Mean Square Error of Prediction, *ASCA *ANOVA-Simultaneous Component Analysis, *SCA *Simultaneous Component Analysis

### Main PMI/burial time-related characteristics of the analyzed biological matrices

As pointed out in Table 5, the studies by Bertoluzza et al. [34] and Woess et al. [35] exhibited consistent trends, indicating a progressive reduction in the organic component in favor of the inorganic fraction over burial time. However, the way this trend was quantified differs between the two studies. Bertoluzza et al. [34] described the phenomenon through the decline of the I₂₉₄₁/I₉₆₀ index, representing the ratio between v_s_ (CH₂) and ν₁ (PO₄³⁻), highlighting the diminishing presence of collagen-associated methylene groups. Conversely, Woess et al. [35] presented this pattern in multiple ways, notably by demonstrating an increase in the ν₄ (PO₄³⁻)/amide I ratio as the PMI advances, reinforcing the progressive mineralization process at the expense of the organic matrix.

Additional variations in inorganic components over burial time were observed in the studies by Baide et al. [36] and Ortiz-Herrero et al. [37]. Baide et al. [36] identified two distinct stages (I and II) of bone decomposition, defining a transition threshold at 42 days based on the intensity of the organic fraction, specifically the carbonate band at 1402 cm⁻¹ (CO₃²⁻). In contrast, Ortiz-Herrero et al. [37] reported spectral modifications occurring over longer periods. They included changes in phosphate bands (ν₂ PO₄³⁻ and ν₄ PO₄³⁻) after 15 years and a further variation in ν₁ PO₄³⁻ between 15 and 50 years. Additionally, spectral shifts in CH₂, amide I, and amide III bands persisted up to 80 years post-burial, indicating prolonged alterations in the organic phase.

The principal component analysis (PCA) carried out by Falgayrac et al. [38] supported the notion that burial time (expressed as X_B_) results in a general reduction of spectral bands primarily associated with organic components, summarized as SC2. However, unlike a strictly linear degradation model, SC1, dominated by ν₁ PO₄³⁻, followed a sinusoidal trend over time. This outline suggests a more complex relationship between burial duration and bone composition, possibly influenced by stabilization phases or secondary processes affecting the bone matrix at different PMI stages.

### Statistical analysis

In the studies conducted by Woess et al. [35], Baide et al. [36], Ortiz-Herrero et al. [37], and Falgayrac et al. [38], multivariate statistical analyses were applied to process and interpret the data obtained from Raman spectroscopy (Table 6). Specifically, the papers of Woess et al. [35], Ortiz-Herrero et al. [37], and Falgayrac et al. [38] referred to Principal Component Analysis (PCA), a mathematical technique used to simplify complex datasets by reducing the number of variables or dimensions while preserving as much of the original variance as possible [40–42]. This method identified new axes in the data space, called principal components, along which the data show the most variation. The first principal component (PC1) accounted for the greatest amount of variance, while the second component (PC2) captured the following greatest variance, in a direction orthogonal (i.e., independent) to PC1. In these three studies, PCA was applied with different purposes. Woess et al. [35] used PCA primarily to reduce the complexity of already visualized data (through chemimaps and MIA – Multivariate Imaging Analysis), identifying the most representative components in a set of 30 Raman spectra – without further detailing them. In contrast, Ortiz-Herrero et al. [37] used PCA only as a preliminary step, aimed at pre-selecting bone samples by identifying and removing outliers, thus reducing sample size. In this case, the PCA was applied to chemical ratios (derived from spectral band intensities) and to mineral crystallinity values. Falgayrac et al. [38], on the other hand, applied PCA within the ASCA framework (ANOVA-Simultaneous Component Analysis), which combines ANOVA and PCA. Unlike Ortiz-Herrero et al. [37], here PCA was used after another preliminary step (ANOVA) to explore data variability patterns once the statistical significance of each factor (X_A_ as source body, and X_B_ as PMI) had been established.

Regarding ANOVA, both Baide and Falgayrac et al. [38] used this method to assess whether a given factor had a statistically significant effect on the dataset. In Baide’s study, ANOVA was applied to test the statistical significance of differences in the integrated area under the 1402 cm⁻¹ Raman band across groups with different PMIs. Once significant differences were confirmed, Tukey’s HSD test was performed to make pairwise comparisons between groups, identifying which ones differed significantly from each other. In Falgayrac’s work [38], ANOVA was used as a preliminary step to PCA. Specifically, after assessing whether the factors X_A_ and X_B_ significantly affected the dataset, PCA was applied to explore how the data varied under the influence of these factors and to detect possible patterns or trends. Among all the studies reviewed, Ortiz-Herrero’s work [37] was the only one to develop a predictive model, using Orthogonal Partial Least Squares Regression (OPLSR). This model was optimized and validated through various statistical metrics, including Root Mean Square Error of Cross-Validation (RMSECV), Root Mean Square Error of Prediction (RMSEP), R², and Q², and achieved an overall prediction accuracy of 71%. The model also allowed identification of the Raman bands most influenced by PMI, indicating the most relevant spectral regions for future applications in PMI estimation.

The application of statistical analyses in the selected studies is summarized and compared in Table 2.

**Table 6:** Statistical analysis performed in the selected studies

Study	PCA	ANOVA	OPLSR
Woess C et al. 2017 [35]	Used to reduce data dimensionality after chemimap and MIA visualization	Not applied	Not applied
Baide A et al. 2020 [36]	Not applied	Applied to evaluate group differences (PMI) based on the area under the 1402 cm⁻¹ band. Tukey HSD used for pairwise comparisons	Not applied
Ortiz-Herrero L et al. 2021 [37]	Applied as a preliminary step for outlier detection based on chemical ratios and mineral crystallinity	Not applied	Developed and validated a predictive model for PMI estimation
Falgayrac G et al. 2022 [38]	Used within ASCA framework, after ANOVA, to explore data variability due to experimental factors	Applied within ASCA framework as a first step to assess the statistical significance of source body and PMI effects before PCA (ASCA)	Not applied

*PMI *Post-Mortem Interval, *MIA *Multivariate Imaging Analysis, *PCA *Principal Component Analysis, *ANOVA *Analysis of Variance, *ASCA *ANOVA-Simultaneous Component Analysis

### Accuracy of Raman spectroscopy-based PMI Estimation

The PMI estimation accuracy was generally acceptable. In the study by Ortiz-Herrero et al. [37], an accuracy deviation < 30% was observed for 71% of specimens; the remaining 29% showed significant inter-subject variability due to the different chemical composition of bone tissue. Falgayrac et al. [38] identified a 15.66% proportion of variance that can be explained by the burial time of skeletal remains, while an additional 7.54% variance was attributed to the source body characteristics.

## Discussion

Our systematic literature review reveals that Raman spectroscopy applications for PMI assessment have been developed mainly in forensic anthropology. Notably, its implementation in this specific field moves from the peak integration of Raman bands to construct models for assessing human skeletal remains’ burial duration or PMI [34, 43]. Biological matrices employed for Raman spectroscopy-based PMI estimation varied from ribs [38], to teeth [34, 36], to long bones such as femur [35] and tibia [37].

Interestingly, a study by Bertoluzza et al. [34] employed exclusively prehistoric remains, including both human and animal samples from Tsurushima Man and Sinomegaceros specimens, respectively. Woess et al. [35] focused on the comparison between forensics and archeological human remains, thus comparing subjects with known, recent time of death with subjects from the Middle Ages.

Indeed, in the case of skeletonized human remains or individual bone findings, although with different time cut-offs from country to country [44–48], it is a priority for judicial authorities to be able to rely on PMI estimations as accurate as possible to distinguish between forensic and archaeological materials.

However, due to complex bone degradation processes, the dating of human skeletal remains is one of the most important and yet challenging tasks of forensic medicine [49]. These degradation processes are known as diagenesis and consist of chemical-physical and biological factors interplaying between bones (with their intrinsic characteristics, such as age, sex, diet, way of life, diseases, and/or medical treatments) [50–52] and the surrounding environment (soil, water, humidity, dryness, gases, suspended particles, pH, microorganisms, and/or cadaver micro- and macrofauna). The complexity and variety of these processes make this degradation not easily predictable [43].

Within this scenario, besides empirical principles based on subjective comparisons and the examiner’s experience [53], several techniques have been applied for the PMI investigation on human skeletal remains. They include macroscopic UV fluorescence [54], radiocarbon tests [55, 56], chemical methods (such as high-performance liquid chromatography-tandem mass spectrometry (HPLC/MS/MS)) [57], microscopic methods [58–60], chemiluminescence analyses (such as the luminol reaction) [45, 46, 53, 61–63], radionuclides’ detection [47, 48, 53, 64–66], proteomics [67, 68], metabarcoding [66, 69], X-ray diffraction (XRD) [44, 70–72], UV-Vis spectroscopy [73–75], UV-Vis-induced fluorescence [76], stereomicroscopy and digital imaging [77], and spectroscopical scans [43, 78], with Fourier transform infrared (FTIR) spectroscopy [35, 49, 75, 79, 80] and Raman spectroscopy already identified as promising techniques for the characterization of bone specimens [43, 81].

When used on skeletal remains, Raman spectroscopy shows specific “fingerprints” due to the presence of organic and inorganic compounds with defined scattering spectra [82].

Various analysis techniques have been proposed, but all of them rely on a multicomponent approach which evaluates the evolution over time of the bone tissue’s main spectral components (PCA). It is based on the understanding of each component’s scattering spectrum, which makes it possible to clearly identify them in the resulting spectra [34–38, 82].

Among the studies selected in this review, some attempted to recreate realistic decomposition processes by re-examining the same remains over a certain time lapse while burying them repeatedly after analysis [38] or leaving them in-field without a burial [36]. Ortiz-Herrero et al. [37], instead, used exhumed remains stored in rooms at known temperature and humidity. In all cases, however, studies have focused on bones without known pathologies and teeth in good conditions (i.e., without cavities or tartar); when in-field preservation was employed, the remains were kept away from wild fauna to avoid damages [36]. Photobleaching was also utilized to reduce the fluorescent background and improve accuracy; the same study also relied on multiple samples acquired from the same specimen in three different, randomly chosen points [37].

In paleo-archeological studies, phosphate re-crystallization evidence was collected and a diminishing trend of relative peaks’ intensity was observed over time [34]. A significant reduction of the protein component in bone remains after the first three years following burial was documented, especially regarding the alpha-helix scattering spectra [82]. Notably, better results for PMI estimation were obtained from the 300–1700 cm^− 1^ region of the spectrum [35].

A study by Baide et al. [36] highlighted significantly different patterns of spectra from day 22 to day 42 after death, suggesting that Raman spectroscopy could be the most accurate technique when employed in cadavers in stage I of decomposition. The same paper showed that vibrational bands did not change in a way that could correlate with the PMI after the first 84 days post-mortem [36]. Moreover, in a real-world-scenario contextualization, Falgayrac et al. [38] proved that bones’ Raman spectra change not only with burial time, but also due to intrinsic factors, i.e., the variability of bone composition. Some studies found differences in spectral bone composition due to gender [83, 84], age [85, 86], pathologies affecting subjects [87], and their medication [88, 89].

Along with these purely analytical limitations, Raman’s translation in forensic practice remains challenging. Indeed, real-life forensic utilization was deemed unfeasible due to the significant differences in terms of both bone composition of the general population and burial conditions [37]. Woess et al. [82] remarked that the combined utilization of Raman spectroscopy and Fourier transform infrared spectroscopy could improve accuracy in PMI estimation, while Bertoluzza et al. [34] suggested an approach with an FT-Raman spectrometer equipped with a Nd: YAG laser.

However, beyond these combined strategies, Raman spectroscopy as a stand-alone technique proved to be a very versatile tool for bone tissue analyses, thanks to its nondestructive nature, microscopic resolution, and ability to provide extensive information about the bone donor (human or nonhuman) [90], the bone mineral and collagen composition, but also a complete examination of bone diagenesis [91–93], which are essential for forensic or anthropological application. Furthermore, Raman spectroscopy has established itself as a valuable tool for determining PMI of bones and teeth by analyzing their evolving chemical alterations over time and correlating them with burial duration [35, 43, 80, 94]. An additional and non-negligible advantage of Raman spectroscopy concerns its possible employment in a portable form [43, 91, 95, 96].

In summary, despite its numerous analytical and practical advantages, the implementation of Raman spectroscopy in forensic practice remains limited. Although the current evidence highlights it as a promising technology, the variability related to samples’ intrinsic characteristics and environmental contexts of preservation affects the widespread adoption and routine use of this technique. Indeed, the low number af available relevant research articles and their heterogeneity in terms of sample size [97] and characteristics (i.e., sex, age, comorbidities), ambient experimental conditions (i.e., soil type and climate), and geographical contexts limit the generalizability of results to any region or population group.

Moreover, further methodological development is needed. The existing literature does not yet provide comprehensive quantitative validation metrics - such as standardized intra- and inter-operator repeatability, inter-instrument and inter-site reproducibility, or a formally characterized total error rate with associated uncertainty bounds - which would be necessary for robust comparison across studies.

From a statistical perspective, our review also shows that most studies rely on exploratory or descriptive approaches (e.g., PCA and ANOVA). In this context, Ortiz-Herrero et al. [14] were the only authors to suggest a predictive OPLS-R model with an accuracy of 71%, which has not yet undergone external validation. The scarcity of robust, externally validated predictive models currently represents one of the main bottlenecks for forensic applications, as it prevents the generalization of results beyond specific laboratory or burial contexts.

Therefore, future research should prioritize both the adoption of standardized quantitative metrics (e.g., ADD) and the development of predictive models with rigorous internal and external validation to enhance the precision and comparability of PMI estimates. These implementations are critical not only from a methodological standpoint, but also to meet Daubert criteria regarding known error rates, reproducibility, and general acceptance, thereby supporting the potential legal admissibility of spectroscopic bone-based PMI estimates.

This gap is consistent with the current absence of meta-analyses on Raman spectroscopy for PMI estimation, as the lack of quantitative indicators contributes to the marked heterogeneity among available studies, making a meta-analytic approach virtually impossible.

A future perspective could aim at including other bands in the spectral analysis. In this context, the Amide II band, which is a collagen-related key component with potential correlation with PMI, may guide future research.

In parallel, the methodological landscape should be broadened by comparing the use of Raman spectroscopy in skeletal remains with alternative approaches such as microRNA (miRNA) profiling and vitreous-humour biochemistry. For example, miRNAs demonstrate considerable promise for PMI estimation due to their post-mortem stability and initial supportive evidence in tissues [98–100]. Meanwhile, biochemical analysis of the vitreous humour (e.g., K^+^, Hx, aminoacids) remains a well-studied thanatochemical method, yet it is affected by environmental and individual variability and the lack of externally validated predictive models [14, 101].

In this context, the Raman spectroscopy-based methods reviewed here offer the advantage of being non-destructive and potentially portable, yet they suffer from the same overarching limitation (absence of robust, generalisable predictive models validated across instruments and sites). Thus, positioning Raman spectroscopy alongside miRNA and vitreous-humour techniques highlights the need to move beyond descriptive correlation towards externally validated predictive modelling, and invites integrated, multimodal studies combining skeletal Raman signals, molecular biomarkers, and fluid biochemistry to improve PMI estimation across a wider temporal and depositional range. Based on all these considerations, the main findings of the present review can be summarized as follows:

- current forensic applications of Raman spectroscopy are mainly of anthropological interest, with bones and teeth being the most commonly investigated biological matrices;
- in the selected studies, the v_1_ (PO_4_)^3-^ peak, corresponding to the ν_1_ symmetric stretching mode of phosphate (an inorganic component of the bone/tooth matrix), is the most frequently evaluated. Carbonate bands are also considered to assess the mineral phase. Instead, the organic component is mainly assessed through collagen-associated bands, with amide ones being the most representative;
- a progressive decrease in the peaks’ intensity of the organic bands, along with an increase in the peaks associated with inorganic bands, is observed with growing PMI. This trend is commonly expressed through the ratio of organic to inorganic band intensities and its variation over time;
- multivariate statistical analyses (e.g., PCA) are employed to enhance the interpretability of Raman spectral data’s variability over PMI;
- Raman spectroscopy’s results are influenced not only by PMI, but also by several intrinsic characteristics of the analyzed matrices (subject’s age, sex, diet, way of life, diseases, and/or medical treatments) as well as by physical-chemical and microbiological features of the burial environment;
- in the context of PMI estimation, no specific Raman applications are so far known to assess early and medium PMI in human cadavers.

## Conclusion

Raman spectroscopy is a fast, non-destructive, versatile, and reliable analytical technique that provides physico-chemical information of virtually any matrix (including biological ones) in any state of matter. In recent years, forensic sciences have demonstrated an increasing interest in this tool due to its invaluable advantages, such as bringing direct analysis results and their easy interpretation with no need for sample preparation, thus qualifying as a time-sparing and cost-effective technique. Other benefits arise from the potential to use portable instrumentations, which can allow on-field and real-time analyses.

Nevertheless, despite its wide range of advantages, there are only a few branches of forensic science that use Raman spectroscopy in practice. Applications in the field of PMI estimation are still limited, being mostly confined to the study of anthropological material.

Contrary to prevailing trends, we wanted to explore the advantages of developing and transitioning this technique to routine forensic practice and especially PMI estimation, which is of importance due to its informative potential within legal and forensic inquiries. With this purpose, the review highlights the need for further laboratory research aimed at implementing Raman spectroscopy for early and medium PMI estimation in human cadavers.
